# Explaining effervescence: Investigating the relationship between shared social identity and positive experience in crowds

**DOI:** 10.1080/02699931.2015.1015969

**Published:** 2015-03-19

**Authors:** Nick Hopkins, Stephen D. Reicher, Sammyh S. Khan, Shruti Tewari, Narayanan Srinivasan, Clifford Stevenson

**Affiliations:** ^a^ School of Psychology, University of Dundee, Dundee, UK; ^b^ School of Psychology and Neuroscience, University of St. Andrews, St. Andrews, UK; ^c^ Centre of Behavioural and Cognitive Sciences, University of Allahabad, Allahabad, India; ^d^ School of Psychology, Queen's University, Belfast, Northern Ireland, UK

**Keywords:** Crowds, Effervescence, Shared identity, Positive emotion, Collective self-realisation

## Abstract

We investigated the intensely positive emotional experiences arising from participation in a large-scale collective event. We predicted such experiences arise when those attending a collective event are (1) able to enact their valued collective identity and (2) experience close relations with other participants. In turn, we predicted both of these to be more likely when participants perceived crowd members to share a common collective identity. We investigated these predictions in a survey of pilgrims (*N* = 416) attending a month-long Hindu pilgrimage festival in north India. We found participants' perceptions of a shared identity amongst crowd members had an indirect effect on their positive experience at the event through (1) increasing participants' sense that they were able to enact their collective identity and (2) increasing the sense of intimacy with other crowd members. We discuss the implications of these data for how crowd emotion should be conceptualised.

One of the most striking features of crowds and mass gatherings is their intense passion (e.g., Ehrenreich, 2006; Durkheim, 1912/1995; Le Bon, 1895/1947). Yet despite this, there is little empirical research or consensus concerning the psychological bases of such emotions (von Scheve & Salmela, 2014). In particular, there has been little research addressing the intensely positive emotional experiences that can characterise collective events. Below, we consider how accounts of group behaviour developed out of the social identity tradition (Tajfel & Turner, 1986; Turner, Hogg, Oakes, Reicher & Wetherell, 1987) can provide a framework for understanding the positive emotions exhibited by crowd participants. We then explore the relationship between perceiving a shared social identity amongst crowd members and experiencing positive emotions using data obtained from pilgrims attending one of the world's largest mass gatherings—the *Magh Mela* at Prayag, Allahabad, India.

## Challenging irrationalism

In his classic text, Le Bon (1895/1947) argued that the emotionality of crowds derives from people losing their sense of self and their capacity for reason: all that remains are the passions. Indeed, Le Bon proposed that the non-conscious contagion of emotions amongst individuals resulted in both an automatic re-alignment of individuals’ behaviour and a consequent loss of control over behaviour (von Scheve & Salmela, 2014). Le Bon's contemporary, Durkheim (1912/1995) agreed on the importance of crowd emotionality and coined an evocative term—*effervescence*—to describe it. Durkheim agreed that emotion could overwhelm crowd members and alter their forms of thought, but he resisted a simple dichotomy between reason and emotion. Indeed, he argued that collective events were socially important contexts in which societal values could be celebrated and re-affirmed. However, while Durkheim rejected the characterisation of collective events as psychologically meaningless, his analysis of the basis for the emergence of strongly positive emotions in the crowd remained vague. Certainly, he offered little analysis of group processes and how they affect the degree to which participants in a collective event are likely to report strongly positive emotional experiences.

More recently, social psychological research has begun to develop a stronger, empirically based analysis of crowd behaviour which more directly challenges Le Bon's assumption that crowd psychology (including the intense emotions associated with participation) involves a distortion of normal functioning. As data on crowd behaviour have accumulated, so the notion that crowd members become mindless and irrationally excitable has been challenged (McPhail, 1991; Turner & Killian, 1972). The social identity approach, which over recent years has come to dominate psychological research into crowd processes (e.g., Reicher, 1987, 2001), also counters the argument that identity and reason are lost in a psychological crowd. Rather, individuals shift from thinking of themselves in terms of their personal identities to thinking of themselves as members of a common category, and the basis for their behaviour shifts from personal belief to group norms. This analysis is supported by historical and psychological evidence that crowd behaviour is meaningful in the sense of reflecting the content of these identities (e.g., Reicher, 1984; Stott & Drury, 2000; Stott, Hutchison, & Drury, 2001; Thompson, 1971). Initially, this focus on the socially meaningful character of crowd action tended to eclipse a consideration of crowd emotion. More recently, however, social identity accounts of the crowd have begun to address the emotional experience of crowds and its antecedents.

## From shared identity to positive experience

Collective emotions can be conceptualised as “common feelings by members of a social unit as a result of shared experiences” (Lawler, Thye, & Yoon, 2014). It is easy for outside observers to assume that those who come together in an event constitute a psychological crowd with shared experiences and common emotions. However, this is misleading. All manner of social divisions may remain relevant even in ostensibly unitary and cohesive events such as national celebrations (Pehrson, Stevenson, Muldoon, & Reicher, 2013) or pilgrimages (Messerschmidt & Sharma, 1981; Sallnow, 1981). These divisions may not only undermine consensus on the experience of the event, but may indeed foster factional and antagonistic social relations.

From a psychological perspective such observations underline the significance of a central tenet of self-categorisation theory's analysis of group behaviour in general (SCT; Turner et al., 1987) and crowd behaviour in particular (Reicher, 1987, 2001). That is, the mere co-presence of a number of people does not constitute a psychological group or crowd. Rather, the formation of a psychological collectivity resides in shared acts of self-categorisation. Research has shown that when there is a shared social identity—that is, when a set of people view themselves and others in terms of a common category membership (e.g., “we are all Americans”)—a number of psychological transformations occur. First, there is a cognitive transformation; people adopt a common frame of reference based on collective norms and values. Those with a shared identity adopt the same perspective based on group beliefs and values, and expect (and seek) agreement with each other (Haslam, Oakes, Reynolds, & Turner, 1999; Haslam & Reicher, 2012; Turner, Oakes, Haslam, & McGarty, 1994). Moreover, a consensual frame of reference serves as a common framework for the evaluation of events and experiences. Second, there is a relational transformation: Those who see others as part of a common “us” become more cooperative and trustful (Tyler & Blader, 2000), more respectful (Renger & Simon, 2011) and more helpful (Levine, Prosser, Evans, & Reicher, 2005; Wakefield et al., 2011) towards each other. Also, people become more comfortable with reduced social and physical distance from fellow in-group members (Alnabulsi & Drury, 2014; Novelli, Drury, & Reicher, 2010; Novelli, Drury, Reicher, & Stott, 2013).

We suggest that both of these transformations can help explain effervescence. First, a shared frame of reference allows for a shared interpretation of events and a common understanding of what contributes to (and undermines) one's experience. In turn, such a frame facilitates coordinated action between crowd members which empowers them to enact their shared values and norms. This ability to enact social identity and behaviorally realise the values associated with one's social identity [what we term *collective self-realisation* (CSR)] is one important source of a positive experience: there is a wealth of ethnographic and interview data, drawn from studies of protestors, to suggest that CSR in crowds is intensely pleasurable and uplifting (Drury & Reicher, 2005, 2009; Drury, Cocking, Beale, Hanson, & Rapley, 2005).

Second, a shared identity brings a sense of connection with others, and this mutual intimacy and warmth in social relationships (what we term *relationality*) is experienced positively. In contrast to the anomie of everyday life—where others are as likely to pass by as to give support, as likely to disagree with one's views as to offer agreement—shared identity in the crowd gives rise to a sense of others as a source of acceptance and recognition. Qualitative evidence illustrates that this sense of relationality, is another basis for deeply positive experience (Neville & Reicher, 2011).

Building on this logic and this earlier work, there is reason to believe that both greater CSR and greater relationality would be associated with positive experience in collective settings. Moreover, there is reason to believe that both CSR and relationality have a basis in the degree to which crowd participants perceive and experience a shared identity with other co-present crowd members. Thus, we predicted that a shared identity amongst crowd members would be associated with crowd members’ positive experience through two routes, CSR and relationality. Or, put slightly differently, we predicted shared identity to have an indirect effect on positive experience *via* both CSR and relationality. In our research, we investigated these proposed associations using survey data from a large collective gathering—a Hindu festival in north India known as the *Magh Mela*.

## The present study

The *Magh Mela* occurs annually and attracts millions of pilgrims to the banks of the Ganges at Prayag (Allahabad, Uttar Pradesh). Many come for three or four days around particularly auspicious bathing days (defined by the lunar calendar) and combine their bathing in the Ganges with shopping and visiting the funfair that borders the site. However, many commit to staying for a full month. These latter (known as *kalpwasis*) subject themselves to a distinctive routine of religious devotion (bathing before dawn and in the afternoon, reciting prayers and attending religious meetings led by *gurus* and *sadhus*). They are typically older than those attending for just a few days (often in their sixties and seventies) and at this later stage of their lives are intent upon seeking religious merit through renouncing all worldly ways and comforts. They live in basic tents and eat one major vegetarian meal a day (without spices which are believed to excite the senses and thus detract from the spiritual). Moreover, the distinctive commitments of the kalpwasis are underlined by the fact that they do not simply commit to live a simple life for the month—they commit to participating in the Mela for 12 consecutive years.

Thus, although at first sight the crowd at the Mela may appear an undifferentiated mass, it is not: the kalpwasis differentiate themselves from non-kalpwasis pilgrims who visit for a few days (Hopkins et al., 2015). Indeed, the former routinely avoid areas of the Mela site occupied by the latter (especially those associated with commerce and the funfair) on the grounds that such experiences subvert the kalpwasis’ goal of renouncing the routine concerns and pleasures of everyday life. Such renunciation is psychologically demanding in the best of environments and is particularly difficult in the cold, noisy and rudimentary temporary camps constructed on the Ganges’ sandy floodplain. Yet, for all this, kalpwasis routinely describe their experience using terms such as *ananda* which translates as “sublime bliss” (Paranjape, 1999; Prayag Magh Mela Research Group, 2007) which comes close to the intensely positive experience associated with the term “effervescence”.

Given that the kalpwasis’ social identity entails relinquishing worldly concerns and devoting oneself to spiritual matters (especially bathing and prayer rituals) it follows that in this context, CSR refers to the extent to which kalpwasis are successful in living a simple spiritual life in which worldly luxuries and concerns are renounced. But this is no easy task. Not only must they endure physical hardship, they must do so in the company of others and this means that these others’ behaviour is not incidental to one's ability to realise and enact the values and ideals of the kalpwasi identity. For example, one of these ideals is that one does not gossip, and if this ideal is to be realised behaviorally it is important that others share and enact it (otherwise, one's own ability to live up to this identity-related ideal would be compromised). So too, if one is to avoid the distractions of argument or non-religious music or argument, it is important that those in one's vicinity do not argue or play such music (Shankar et al., 2013).

As kalpwasis are easily distinguishable (e.g., by their living area and routines) and differentiate themselves from non-kalpwasis attending the Mela, there is a basis for kalpwasis seeing each other as part of a single group (Prayag Magh Mela Research Group, 2007) and for mutual support and social influence (Pandey, Stevenson, Shankar, Hopkins, & Reicher, 2014). However, this cannot be assumed (Messerschmidt & Sharma, 1981). Not only are there different groups from different regions, following different gurus and traditions, but more mundane tensions associated with collective living may subvert a sense of shared identity.

Accordingly, we expected variation in the degree to which kalpwasis reported a sense of shared identity, and that higher levels would be associated with a more positive kalpwasi experience. Moreover, we expected the association between shared identity and positive experience to be mediated by two processes. First, as a shared identity entails a common frame of reference for the interpretation of events and an alignment of values and purpose, we expected higher levels of shared identity to be associated with greater CSR. Second, we expected higher levels of shared identity to be associated with greater “relationality” (i.e., more intimate social relations). Accordingly, we predicted that the greater the perception of a shared identity, the more intensely positive the individual's experience and that this relationship would be mediated *via* both CSR and relationality (in parallel).

## METHOD

### Sample

Data were obtained through an orally administered questionnaire delivered to 416 kalpwasis attending the 2011 Mela (*M*_age_ = 64.38 years, *SD* = 9.32 years). Two-hundred and thirty-seven (57.0%) were female. Three-hundred and eighty-four (92.3%) belonged to the General Caste (GC) category and 32 (7.7%) to the Other Backward Caste (OBC) category. This age, gender and caste profile is representative of the demographic profile of the broader kalpwasi population. One-hundred and seventy-seven were illiterate (42.5%), 192 (46.2%) held primary-to-intermediate education and 47 (11.3%) were university educated.

### Procedure

The measures were translated and back-translated (English–Hindi–English) and piloted amongst Hindi-speaking participants with various educational backgrounds. The questionnaires were administered by a trained team of field investigators and took approximately 30 minutes to complete. When approaching potential participants, the researchers gave an overview of the questions to be asked. Because of literacy issues, participants gave oral informed consent (this was approved by the Ethics Committees of the Universities of Dundee and Allahabad). As a sample from rural India has little (if any) experience of questionnaire surveys, and still less experience of using 5-point scales, we explained how participants could communicate their answers using drawings of five glasses containing increasing levels of water (ranging from empty to full; Tewari, Khan, Hopkins, Srinivasan, & Reicher, 2012).

### Measures

The questionnaire used in this research and all our data are publicly available (http://data-archive.ac.uk). The questionnaire contained items not relevant to the research reported here (e.g., concerning participants’ health; Khan et al., in press a, in press b). The scales relevant to our research question are documented below. For each scale, we report an indicative item. The full set of items for each scale is reported in Table 1. Responses to each item were obtained on 5-point scales (anchored: 1 = *not at all*; 5 = *completely* which translates conceptually as *a lot*) and were averaged to calculate overall scale scores for each participant. Each scale was positively scored.

**Table 1.** Scale items: loadings and cross-loadings

	Factors			
Items	1	2	3	4
Factor 1: relationality				
To what extent do other Kalpwasis:				
1. demonstrate feelings of love to you?	.83	(.29)	(.23)	(.35)
2. help you when you need it?	.83	(.30)	(.25)	(.44)
3. behave towards you in a respectful manner?	.83	(.19)	(.18)	(.40)
4. behave towards you with understanding of your needs as a Kalpwasi?	.82	(.25)	(.17)	(.36)
5. behave towards you in a way that allows you to fulfil your Kalpwasi?	.75	(.26)	(.21)	(.39)
Factor 2: positive experience				
In the period of Kalpwas:				
1. to what extent have you felt happier than you have ever felt in your life?	(.26)	.87	(.20)	(.17)
2. to what extent have you felt more fulfilled than you have ever felt in your life?	(.26)	.86	(.32)	(.18)
3. to what extent have you felt better than you have ever felt in your life?	(.23)	.85	(.16)	(.12)
4. to what extent have you felt more contended than you have ever felt in your life?	(.28)	.80	(.25)	(.14)
5. to what extent have you felt more alive than you have ever felt in your life?	(.30)	.79	(.36)	(.15)
Factor 3: collective self-realisation				
In the period of Kalpwas, to what extent do you feel:				
1. you are able to completely ignore the everyday concerns of this world to concentrate on the spiritual?	(.15)	(.22)	.80	(.26)
2. you are able to fully live a simple life in accordance with religious teaching?	(.21)	(.28)	.76	(.27)
3. you are able to fully overcome the restrictions of everyday life and live in accordance with your religious faith?	(.09)	(.15)	.76	(.23)
4. you are able to totally devote yourself to following religious scriptures?	(.27)	(.25)	.73	(.25)
5. you feel you are able to fully devote yourself to performing your religious rituals?	(.27)	(.24)	.69	(.22)
Factor 4: shared identity				
To what extent do you think that all Kalpwasis:				
1. think of themselves as part of one large family?	(.37)	(.17)	(.24)	.89
2. have a sense of “we-ness” with other Kalpwasi?	(.42)	(.18)	(.28)	.84
3. have a feeling of unity amongst each other?	(.42)	(.22)	(.30)	.76
4. besides their differences, share the same identity?	(.41)	(.12)	(.22)	.73
5. think of themselves as part of a single group?	(.27)	(.07)	(.23)	.70

*Shared identity*: five items measured the extent to which kalpwasis saw themselves as constituting a collective (e.g., *To what extent do you think that all kalpwasis think of themselves as part of a single group?*).

*Relationality*: five items measured the extent to which participants perceived their interactions and relations with other kalpwasis to be intimate (e.g., *To what extent do other kalpwasis behave towards you in a respectful manner?*).

*Collective self-realisation*: five items measured the extent to which participants believed they were able to enact the ideal Hindu identity during the Mela (e.g., *In the period of kalpwas, to what extent do you feel you are able to fully live a simple life in accordance with religious teaching?*).

*Positive experience*: five items measured the extent to which participants judged their experience of participating in the Magh Mela to be uniquely positive (e.g., *In the period of kalpwas, to what extent have you felt more fulfilled than you have ever felt in your life?*).

### Scale properties

We examined the dimensionality of our variables using principal axis factoring (PAF) (Oblimin rotation with Kaiser Normalisation) which is particularly appropriate when the scales are new (inevitable given our research location). The results revealed the items loaded onto four discrete factors with eigenvalues greater than one and that these corresponded to our four pre-defined measures. The four factors explained 64.23% of the total item variance. Table 1 reports the factor loadings and cross-loadings for all the items. Table 2 presents these scales’ Cronbach's alphas, their scale means and standard deviations, and their inter-scale correlations. The reliabilities of all measures were excellent. The measures correlated with one another moderately.

**Table 2.** Scale means, standard deviations, Cronbach's alphas and inter-scale correlations

	Shared identity		Relationality		Collective self-realisation		Positive experience	
	M (SD)	α	M (SD)	α	M (SD)	α	M (SD)	α
	4.54 (.61)	.88	4.73 (.45)	.91	4.43 (.59)	.86	4.79 (.44)	.92
			*r*		*r*		*r*	
Shared identity			.44***		.30***		.18***	
Relationality					.24***		.30***	
Collective self-realisation							.29***	

*** *p* < .001.

## RESULTS

In order to address our hypotheses we employed hierarchical regression modelling. Specifically, we examined whether shared identity, relationality and CSR predicted positive experience (whilst controlling for the effects of the participants’ age, gender, caste, marital status and education). The variables were entered in four steps: (1) age, gender, caste and marital status; (2) education; (3) shared identity; (4) relationality and CSR. The results are presented in Table 3.

**Table 3.** Hierarchical regression model: predicting positive experience

	Step 1			Step 2			Step3			Step 4		
Model	B	SE	β	B	SE	β	B	SE	β	B	SE	β
1. Age	−.00	.00	−.07	−.00	.00	−.07	−.00	.00	−.06	−.00	.00	−.04
Gender	.07	.05	.08	.05	.06	.05	.07	.06	.08	.05	.06	.06
Caste	.07	.08	.05	.07	.08	.04	.06	.08	.05	.05	.06	.06
Marital status	−.07	.05	−.07	−.08	.05	−.07	−.10	.05	−.09	−.09	.05	−.08
2. Primary-to-intermediate				.04	.06	.04	.03	.06	.04	.04	.05	.04
University				.05	.09	.03	.06	.09	.04	.02	.09	.02
3. Shared identity							.14	.04	.20***	.02	.04	.02
4. Relationality										.23	.05	.23***
Collective self-realisation										.17	.04	.23***
Adjusted *R*^2^	.00			−.00			.03			.13		
Δ *∆R*^2^	*F*(4, 411) = 1.26			*F*(2, 409) = .21			*F*(1, 408) = 15.98***			*F*(2, 406) = 23.78***		
ANOVA	*F*(4, 411) = 1.26			*F*(6, 409) = .91			*F*(7, 408) = 3.09**			*F*(9, 406) = 7.97***		

** *p* < .01; ***p < .001.

The adjusted *R* ^2^ and the *R* ^2^ change values were only significant at the third and fourth steps of the analysis, indicating that the socio-demographic characteristics of the participants did not exert a significant influence upon positive experience. Shared identity was a significant predictor at the third step. However, it was non-significant when relationality and CSR were entered together (at the fourth step). Moreover, at this fourth step both relationality and CSR were significant predictors of positive experience. Overall, the model explained 13% of the variance in positive experience.

That shared identity was a significant predictor of positive experience at the third step but not the fourth is compatible with the idea that its association could be indirect *via* relationality and CSR. This was explored in an analysis of indirect effects (Hayes, 2012) in which shared identity was entered as the independent variable, positive experience as the dependent variable, relationality and CSR as mediators (in parallel), and the socio-demographic variables as covariates. To avoid multicollinearity the variables were standardised.

The analysis (95% confidence intervals, based on 5000 bootstrap samples) revealed the total effect of shared identity on positive experience was positive and significant (*total effect* = .0907, 95% CI: .0478, .1337). More importantly, when relationality and CSR were entered (simultaneously) as parallel paths from shared identity to positive experience, the results indicated significant indirect effects via both relationality (*indirect effect* = .0470, 95% CI: .0241, .0795) and CSR (*indirect effect* = .0295, 95% CI: .0152, .0516). Consistent with the hierarchical regression analysis, when these two paths were taken into account, the direct effect of shared identity on positive experience was non-significant (*direct effect* = .0142, 95% CI: −.0329, .0614). Thus, these analyses suggest that shared identity had indirect effects on positive experience *via* CSR and relationality. Repeating the analysis using non-standardised scales confirmed these results.

Given the potential for more complex relationships between these variables we conducted further analyses. One set concerned the relationship between CSR and relationality. It is theoretically possible that greater relationality would be associated with greater CSR, either because relationality is itself an antecedent of CSR (positive relations facilitating CSR), or because CSR is an antecedent of relationality (such that the experience of CSR leads one to experience more intimate relations with others). We investigated these more complex accounts of how shared identity was associated with positive experience in two analyses of (serial) indirect effects. The first tested whether shared identity had an indirect effect on positive experience through relationality and then from this, through CSR. Investigating this (serial) sequence using PROCESS (Hayes, 2012) showed the total effect of shared identity on positive experience was as before (*total effect* = .0907, 95% CI: .0478, .1337), and that shared identity had an indirect effect on positive experience *via* relationality and then CSR (serially) (*indirect effect* = .0069, 95% CI: .0020, .0153). The direct effect of shared identity on positive experience was non-significant (*direct effect* = .0142, 95% CI: −.0329, .0614). In other words there was an effect of shared identity on relationality, and then, through this, an effect on CSR which then predicted positive experience. The second analysis tested whether shared identity had an indirect effect on positive experience *via* CSR and then relationality. Again, we found evidence for this (serial) indirect effect of shared identity on positive experience (*indirect effect* = .0035, 95% CI: .0007, .0098). However, in evaluating the significance of these more complex (serial) pathways it is clear that though significant, they are modest. The same results were obtained using non-standardised variables.

We also reasoned there could be a degree of bi-directionality in these variables’ relationships. For example, a sense of CSR may lead one to identify more strongly with co-present others (that is, a sense of shared identity could arise from a sense of CSR). In similar vein, greater relationality may facilitate a shared identity because one infers a commonality on the basis of one's more intimate social relations. Again, we investigated these reciprocal relationships using serial indirect effects. First, we investigated whether CSR had an indirect effect on positive experience through shared identity and relationality (serially). The total effect of CSR on positive experience was significant (*total effect* = .1292, 95% CI: .0880, .1703) and analysis confirmed an indirect effect of CSR on positive experience *via* shared identity and relationality (serially) (*indirect effect* = .0122, 95% CI: .0057, .0228). Again, this latter effect was modest and the direct effect of CSR on positive experience remained significant (*direct effect* = .1009, 95% CI: .0587, .1430). Second, we investigated if relationality had an indirect effect on positive experience *via* shared identity and CSR (serially). The total effect of relationality on positive experience was significant (*total effect* = .1298, 95% CI: .0887, .1709) as was the indirect effect of relationality on positive experience *via* shared identity and CSR (serially) (*indirect effect* = .0104, 95% CI: .0044, .0203). Again this serial indirect effect was modest and the direct effect of relationality on positive experience remained significant (*direct effect* = .0984, 95% CI: .0528, .1441). The same results were obtained using non-standardised variables. Taken together these last two analyses suggest that there is potential for people's sense of relationality and CSR to contribute to a sense of shared identity and hence impact upon positive experience. However, whilst these reciprocal paths may be significant, their effects are small.

Thus far, we have presented analyses which treat positive experience as an outcome variable of identity-related processes. However, it is important to note that emotions in a crowd may shape group processes. For example, it is possible that a positive experience could encourage identification with others which in turn predicts CSR and relationality. We investigated this in two separate analyses of (serial) indirect effects. First, we considered whether participants’ positive experience was associated with CSR *via* shared identity and relationality (serially). Second, we tested whether positive experience was associated with relationality *via* shared identity and CSR (serially). For both models the total effects of positive experience were significant (first model *total effect* = .1734, 95% CI: .1182, .2286; second model *total effect* .1330, 95% CI: .0910, .1751). However, neither analysis found evidence for the postulated serial indirect effects (first model *indirect effect* = .0044, 95% CI: −.0006, .0138; second model *indirect effect* = .0016, 95% CI: −.0001, .0054) and both direct effects remained significant (first model *direct effect* = .1344, 95% CI: .0782, .1905; second model *direct effect* = .0861, 95% CI: .0462, .1260). We also investigated whether positive experience mediated the effects of relationality and CSR on shared identity. Specifically, we investigated whether CSR had an indirect effect on social identity *via* relationality and positive experience (serially), and whether relationality had an indirect effect on shared identity *via* CSR and positive experience (serially). For both these models the total effect of positive experience were significant (first model *total effect* = .1731, 95% CI: .1168, .2293; second model *total effect* = .2819, 95% CI: .2301, .3337). Neither analysis found evidence for the postulated serially mediated indirect effects (first model *indirect effect* = .0010, 95% CI: −.0018, .0064; second model *indirect effect* = .0010, 95% CI: −.0015, .0060) and both direct effects remained significant (first model *direct effect* = .1063, 95% CI: .0522, .1604; second model *direct effect* = .2508, 95% CI: .1967, .3049). Needless to say, whereas this suggests that our variables help predict positive experience when that is the outcome variable of interest, it is inappropriate to make strong unilateral claims about causal direction on such data (see below).

## DISCUSSION

Our findings show that participants’ level of relationality and CSR were associated with the positivity of their emotional experience at the event. Moreover, both relationality and CSR were associated with the degree to which our kalpwasi participants believed there was a common identity amongst kalpwasi pilgrims. Indeed, we found that perceptions of a shared identity had an indirect effect on positive experience *via* both relationality and CSR (in parallel). However, we also found evidence for more complex associations between shared identity and the positivity of emotional experience. First, relationality and CSR functioned as serial mediators of the shared identity–positive experience relationship. Second, when predicting positive experience we found evidence for a degree of bi-directionality in the relationship between CSR, relationality and shared identity.

These quantitative data should be interpreted alongside the ethnographic and interview data obtained with kalpwasis (Hopkins et al., 2015, Pandey et al., 2014; Shankar et al., 2013). As concerns the experience of relationality, interviewees reported how they came together and had a sense of support and concern from fellow pilgrims in the Mela (in contrast to other collective settings like the railway station where they lack shared identity). They also reported the pleasure derived from knowing others would support them. For example, one (cited in Hopkins et al., 2015) explained:

in the home, or in villages, people see each other and feel tensions [excluded material] But here, so much of goodness comes in people, even more than at home [excluded material] someone will say “come sister sit, warm up your hands and feet” [excluded material]. It feels good. And there [back home], if you interact more, even in a family, the close relatives cannot stay along with each other!

As concerns the experience of CSR, interviewees spoke of the pleasures of being able to realise the injunction to behave in a spiritually pure way and of how this was facilitated by a shared identity. Thus, to draw another example from our work, one kalpwasi (cited in Hopkins et al., 2015) referred to the concept of *satsang* (which translates as an assembly of persons who listen to and speak spiritual truths) and explained the pleasure to be found in being part of a collective in which spiritual values could be expressed and enacted:

the most important thing here is the *satsang*. No one gossips about others. No one wants or looks for failings in others [excluded material]. All become like one family. This is what is called *satsang*. Kalpwas means this only—that you do not criticize or gossip about each other. Each one follows the rules.

We do not suggest our investigations explain everything about positive experience in the crowd. As we have already intimated, all sorts of physical and practical factors may be of relevance. This event poses many physical challenges (Pandey et al., 2014; Shankar et al., 2013). The weather can be cold, wet and miserable; people fall ill; one's tent and sanitary conditions may be poor; it is often very noisy, etc. In this context, the fact we can account for 13% of the variance is striking and it may be addressing much of the specifically psychological determinants of positive experience. With regard to this latter, it should be noted that our questionnaire scale items did not just refer to feeling positive, but to feeling more positive than one ever has previously in one's life. This suggests that we are getting close to the positive passion—the “effervescence”—which has so struck observers of the crowd.

As to what can be inferred from these data there are a number of issues. Throughout, we have conceptualised positive experience as the outcome of identity-related processes. However, several caveats are in order. One is methodological: our data are cross-sectional and this constrains our ability to make strong claims about the causal ordering of our predictors. Another caveat is conceptual: as Blumer (1939) acknowledged long ago, crowd processes are likely to be circular in nature and it easy to imagine a feedback loop in which the various elements of our model contribute towards a spiralling of emotional intensity. Thus, just as a shared identity may contribute to a sense of relationality, so this latter may be a basis for participants to infer a sense of shared identity. Also, just as a positive experience may be a product of CSR and relationality, so the positivity of one's experience may contribute to the experience of relationality and CSR. For example, we know displays of emotion can be communicative and the sharing (actual or presumed) of an emotional state may facilitate affiliation with others (Hess, Houde, & Fischer, 2014). Nevertheless, given our specific focus in this paper on the antecedents of positive experience, our research highlights the utility of considering shared identity as a key predictor and as having indirect effects on positive experience *via* relationality and CSR (in parallel). This does not rule out (indeed it encourages) future research into the consequence of positive experiences in groups and crowds.

Another caveat concerns the generalisability of these associations: What can a study of rural Indians at a Hindu religious festival tell us about positive crowd experience more generally? In response it should be noted that religious social identifications are of enormous social and political significance in everyday life and to say that a phenomenon is “only” relevant to religion does not diminish its significance. Indeed, Durkheim himself studied effervescence in religious gatherings precisely because he believed that these encapsulate processes that are common to all collective functioning (Olaveson, 2001). Moreover, many researchers investigating the effects of religious participation suggest that these may derive from the general experience of being part of a group (or congregation) rather than from specifically religious beliefs (Graham & Haidt, 2010; Ysseldyk, Matheson, & Anisman, 2010). More generally, despite the limitations of correlational and cross-sectional research such as ours, it is important not to overlook the value of complementing experimental research with survey research conducted in field settings. Indeed, it could be added that there is particular value in investigating these relationships in a field-setting in north India: the generality of any theory developed and tested in the urban West depends on showing that similar processes operate amongst very different populations.

Nonetheless, if there is reason to believe that our constructs may have more general applicability, it is important to recognise that the precise form that these take may vary from collectivity to collectivity as a function of specific norms and values. This is most obvious in the case of CSR (which explicitly denotes the enactment of group identity). In the case of kalpwasis this entails spiritual immersion. At other events (e.g., a rock festival), it will entail different behaviours. Indeed, in studies of demonstrations and riots, CSR often involves obstruction and violence that imposes the collective's will on an antagonistic out-group, notably the police (Drury & Reicher, 2005). In the Mela, the identity and hence the behaviours relevant to CSR are very different, and to investigate whether our reasoning applies in any given collectivity it is necessary to be sensitive to local cultural matters as well as to general psychological processes.

More definitive conclusions about generality must await a body of research looking at different types of collective events involving different groups. But, in the case of Prayag's *Magh Mela* we can say with some confidence that the intense joy expressed by crowd members fits better with Durkheim's analysis than Le Bon's. Effervescence does not denote mindlessness. Rather it is associated with the sense that one is able to live by one's shared beliefs and from the close bonds that are forged with others. A shared social identification with others makes both more likely. By specifying the intervening processes more precisely and by testing them more systematically than before, we hope to have gone some way towards unpacking the age-old puzzle of the psychological bases for positive collective emotion.
